# Supplementary material for: The microRNA cluster miR-183/96/182 contributes to long-term memory in a protein phosphatase 1-dependent manner
Source: Nat Commun. 2016 Aug 25;7:12594. doi: 10.1038/ncomms12594 (PMC5007330; doi:10.1038/ncomms12594)
Supplement: Supplementary Information — Supplementary Figures 1 - 17 and Supplementary Tables 1 and 2 [file ncomms12594-s1.pdf]

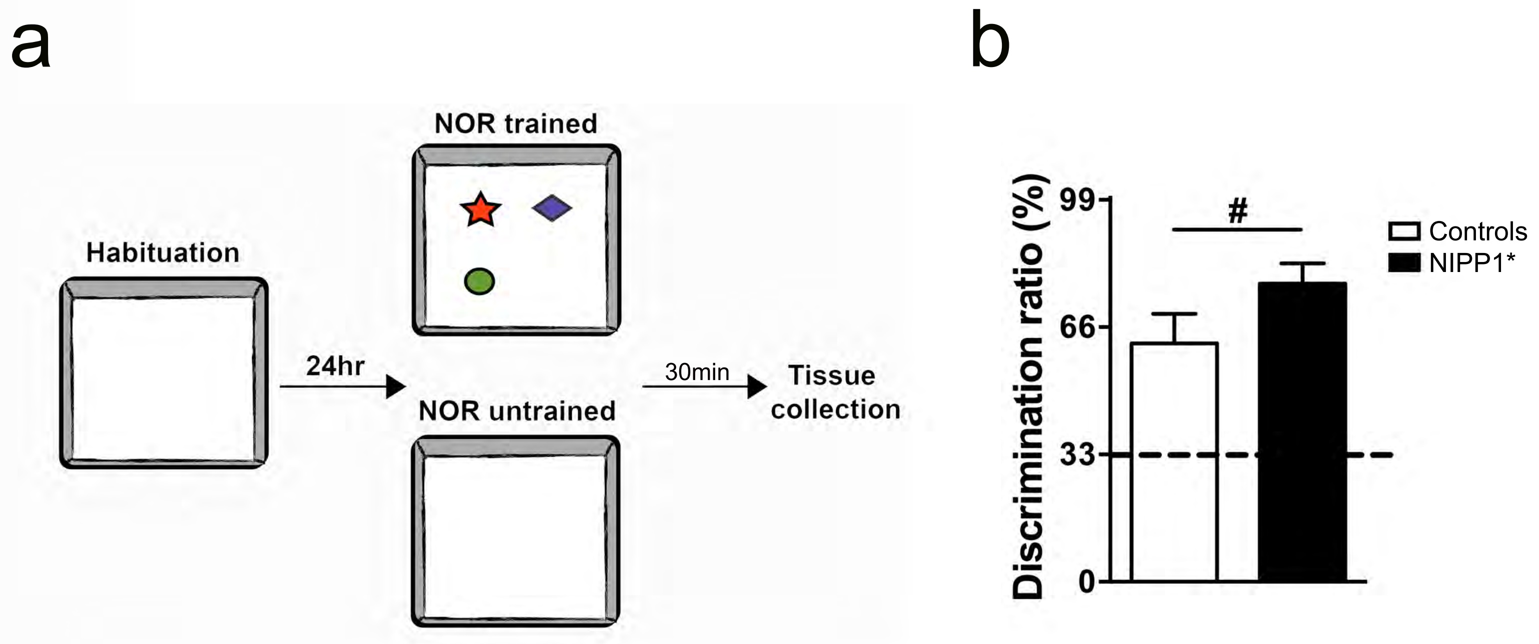


Supplementary Figure 1: NOR training of mice before deep sequencing screening. (a) Experimental set-up for NOR training conducted prior to deep sequencing. (b) Performance of animals used for sequencing tested 5min after training, expressed as discrimination ratio. Both groups demonstrated significant discrimination of novel object (chance level set at 33%) (one-sample t-test, control: $t_5=4.589$, $p<0.01$; NIPP1*: $t_5=8.525$, $p<0.001$) but NIPP1* mice had better performance than controls (unpaired t test between controls and NIPP1*: $t_{10}=1.93$, #$p=0.08$). Controls, n=6; NIPP1*, n=6. Bar graphs represent mean ± s.e.m.

a

| Insert range | Matching to genome (%) | Matching to miRNA (%) |
|---|---|---|
| 19 - 26 | 96.9 | 92.1 |
| 19 - 44 | 94.7 | 82.9 |
| 27 - 44 | 66.3 | 0.1 |

b

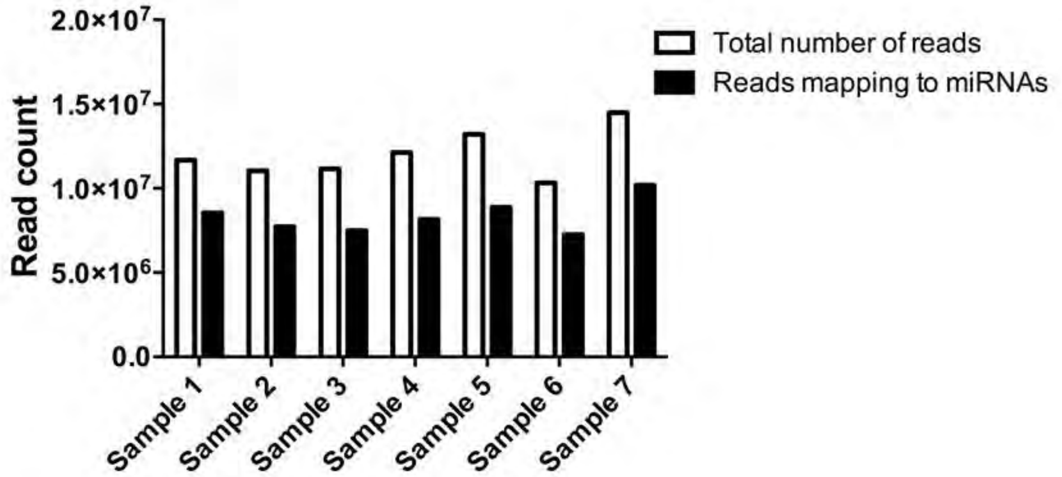


c

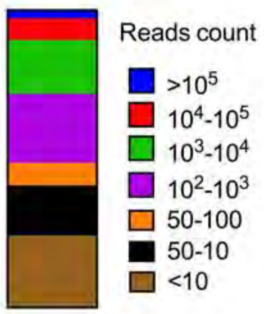


d

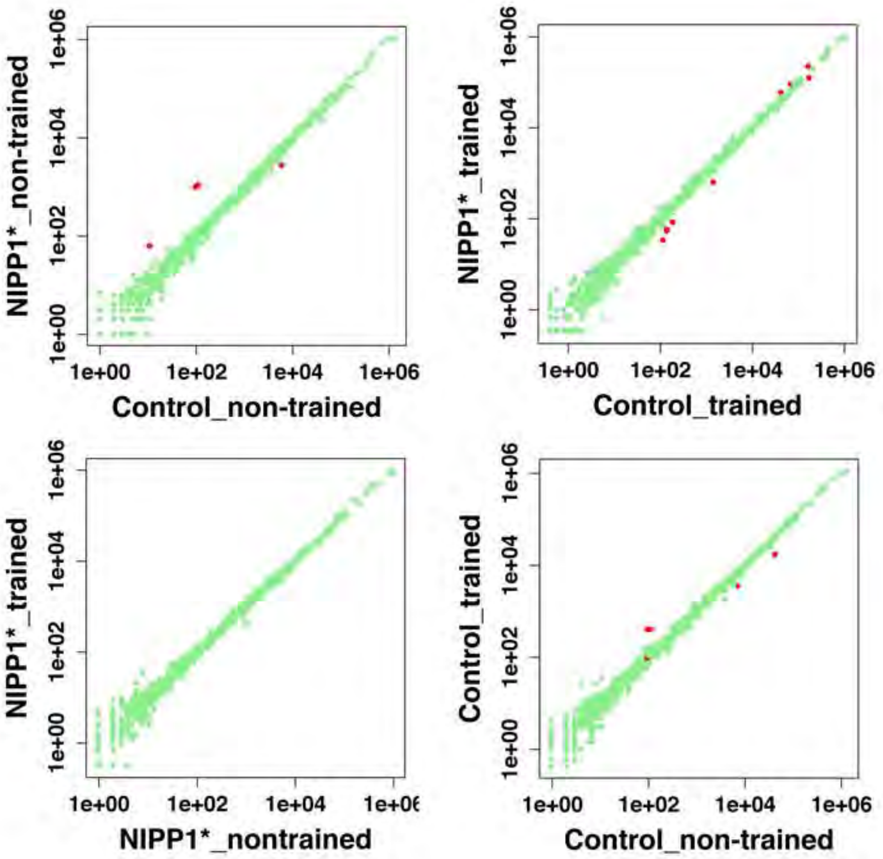

Supplementary Figure 2: Distribution of reads obtained by deep sequencing and correlation between groups. (a) Proportion of sequence reads mapping to the genome and known miRNAs. (b) Comparison of number of reads that uniquely map to miRNAs in each sample (c) Average distribution of all identified miRNAs based on reads count expressed as ‘read counts’. (d) Correlation of expression level of all identified miRNAs (each represented as a dot in ln(read count)) ) between the different experimental groups (NIPP1* and control littermates, trained or untrained). Red dots represent differentially expressed miRNAs (adjusted $p<0.05$).

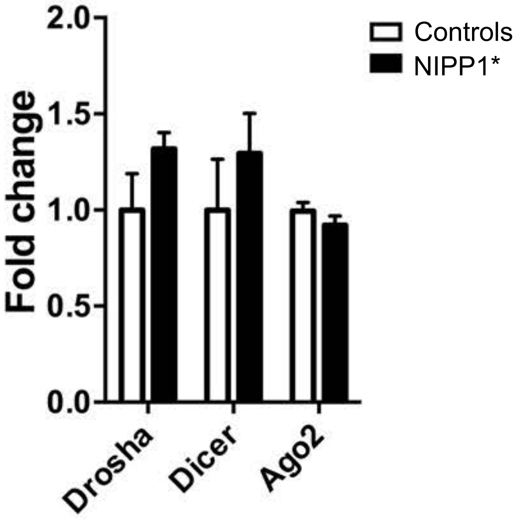


Supplementary Figure 3: qPCR quantification of major components of the miRNA biogenesis machinery in NIPP1* and control mice. Drosha: $t_8=1.54$, $p=0.16$; Dicer: $t_8=0.89$, $p=0.40$; Ago2: $t_8=1.10$, $p=0.30$; Controls, n=5; NIPP1*, n=5. Bar graphs represent mean ± s.e.m.

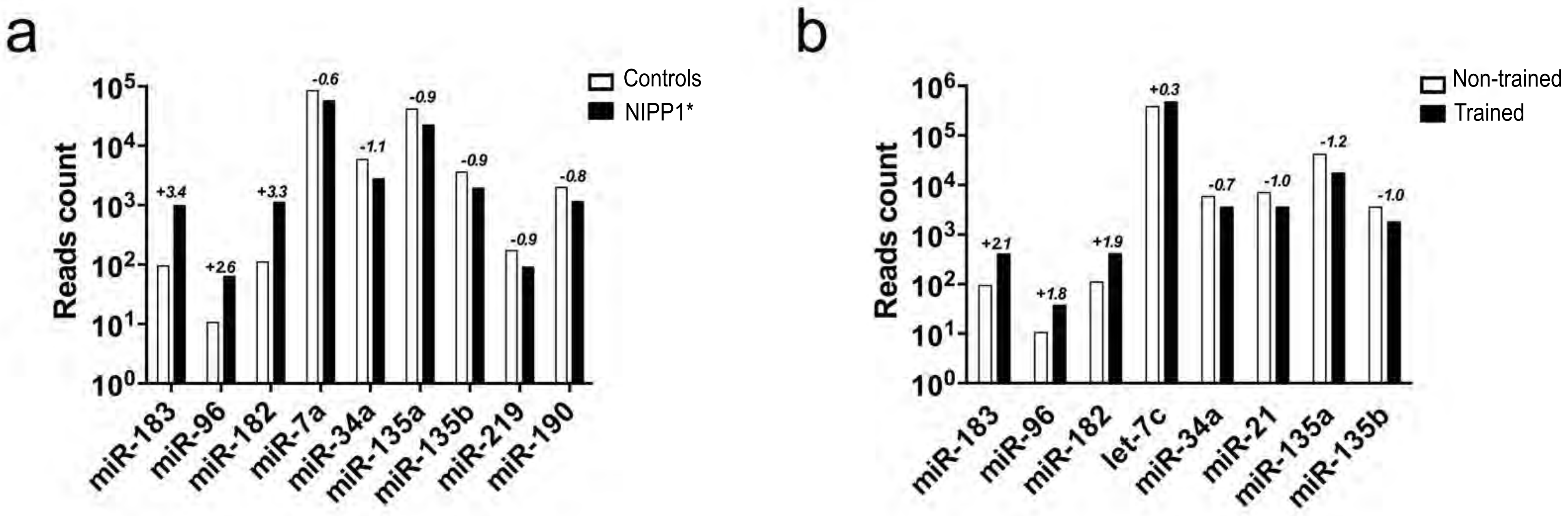


Supplementary Figure 4: Relative abundance of differentially expressed miRNAs based on reads count per million (log 2). (a) NIPP1* compared to control mice; (b) trained controls compared to non-trained controls.  The number above bars indicates fold change between groups.

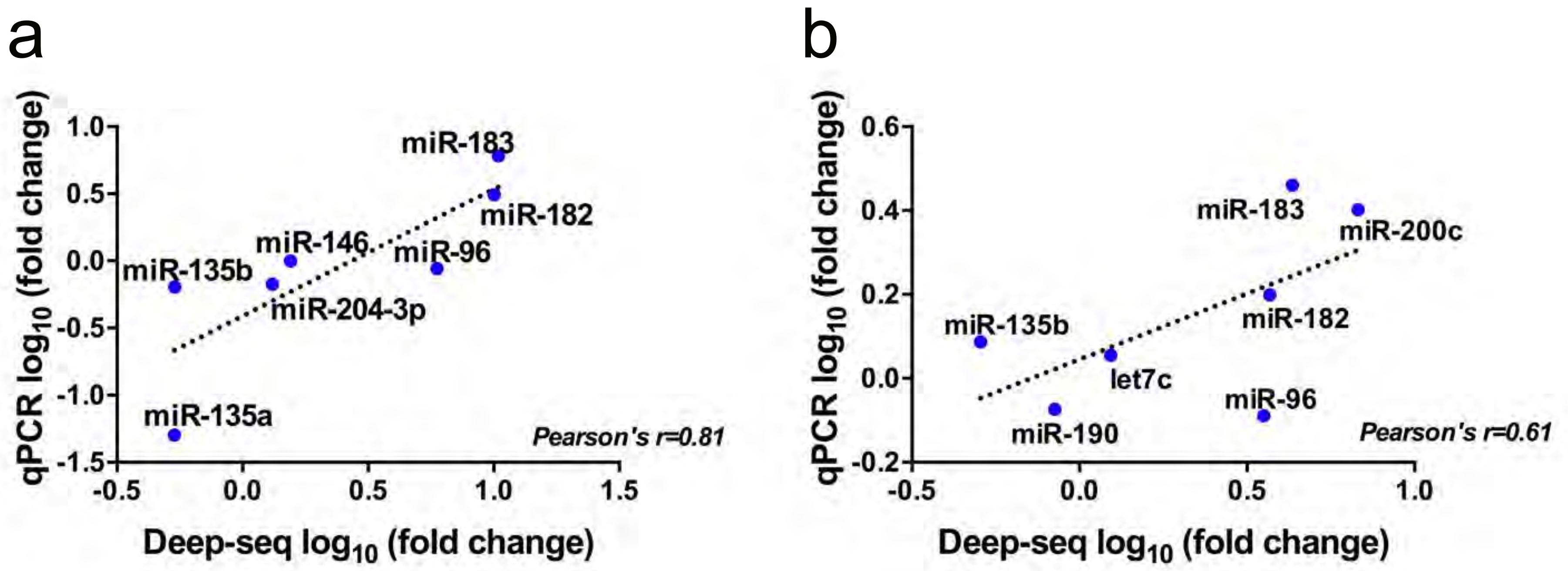


Supplementary Figure 5: Correlation between deep sequencing and qPCR-based quantification of miRNAs differentially expressed: (a) in NIPP1* and control mice; (b) in NOR-trained and untrained mice.

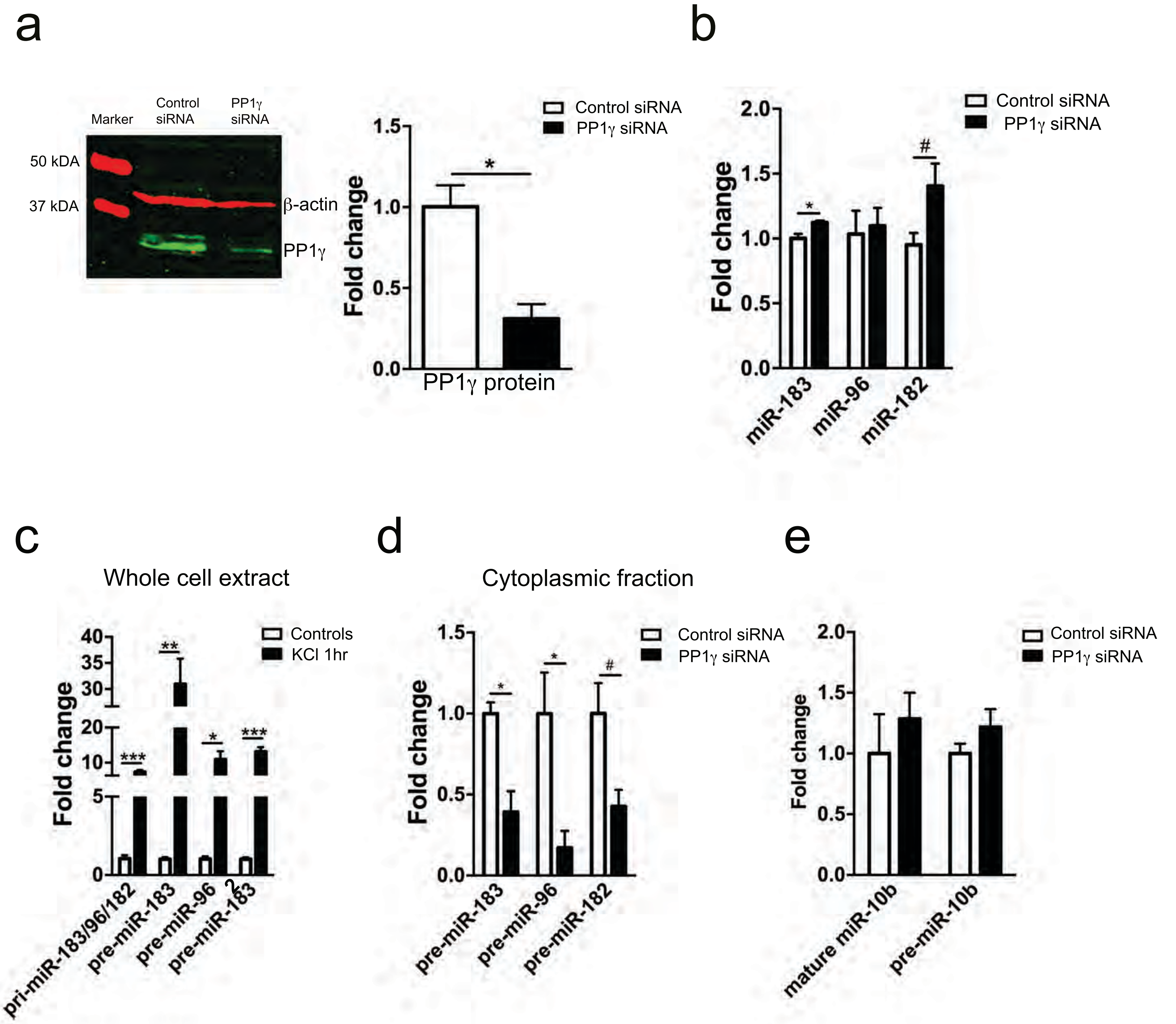

a
Marker
Control siRNA
PP1γ siRNA
50 kDA
37 kDA
β-actin
PP1γ
Control siRNA
PP1γ siRNA
Fold change
PP1γ protein
b
Control siRNA
PP1γ siRNA
Fold change
miR-183
miR-96
miR-182
c
Whole cell extract
Controls
KCl 1hr
Fold change
pri-miR-183/96/182
pre-miR-183
pre-miR-96
pre-miR-183
d
Cytoplasmic fraction
Control siRNA
PP1γ siRNA
Fold change
pre-miR-183
pre-miR-96
pre-miR-182
e
Control siRNA
PP1γ siRNA
Fold change
mature miR-10b
pre-miR-10b

Supplementary Figure 6: Effect of PP1γ inhibition on miR-183/96/182 precursor and mature transcripts in N2A cells. (a) Representative Western blot of PP1γ in whole cell extracts of N2A cells transfected with PP1γ siRNA (left panel), and quantification of blots (right panel, $t_4=4.31$, *$p<0.05$). (b) Mature miR-183/96/182 expression in N2A cells (whole cells fraction) afterPP1γ knockdown (miR-183: $t_4=3.26$, *$p<0.05$; miR-96: $t_4=0.28$, $p=0.8$; miR-182: $t_4=2.33$, #$p=0.08$). (c) Pri-miR-183/96/182 and pre-miR-183/96/182 expression in whole cell extract of N2A cells after 1hr KCl stimulation (pri-miR-183/96/182: $t_4=4.31$, ***$p<0.001$; pre-miR-183: $t_4=6.13$, **$p<0.01$; pre-miR-96: $t_4=4.20$, *$p<0.05$; pre-miR-182: $t_4=9.26$, ***$p<0.001$).
(d) Cytoplasmic pre-miR-183/96/182 level in N2A cells after PP1γ knockdown and 1hr KCl stimulation (pre-miR-183: $t_4=4.16$, *$p<0.05$; pre-miR-96: $t_4=3.02$, *$p<0.05$; pre-miR-182: $t_4=2.68$, #$p=0.06$). (e) Level of mature ($t_4=1.29$, $p=0.27$) and precursor ($t_4=0.73$, $p=0.50$) transcripts of miR-10b in cells treated with PP1γ siRNA. Bar graphs represent mean ± s.e.m.

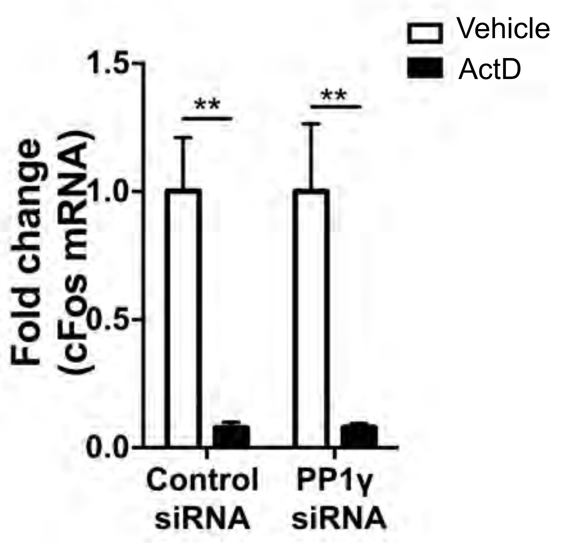


Supplementary Figure 7: Verification of Actinomycin D treatment on c-Fos expression in N2A cells treated with KCl for 1hr; (Two-way ANOVA, ActD-$F_{1,8}=29.77$, $p=0.0006$. post-hoc: Control siRNA $t_8=3.86$, $^{**}p<0.01$; PP1γ siRNA: $t_8=3.86$, $^{**}p<0.01$). Bar graphs represent mean ± s.e.m.

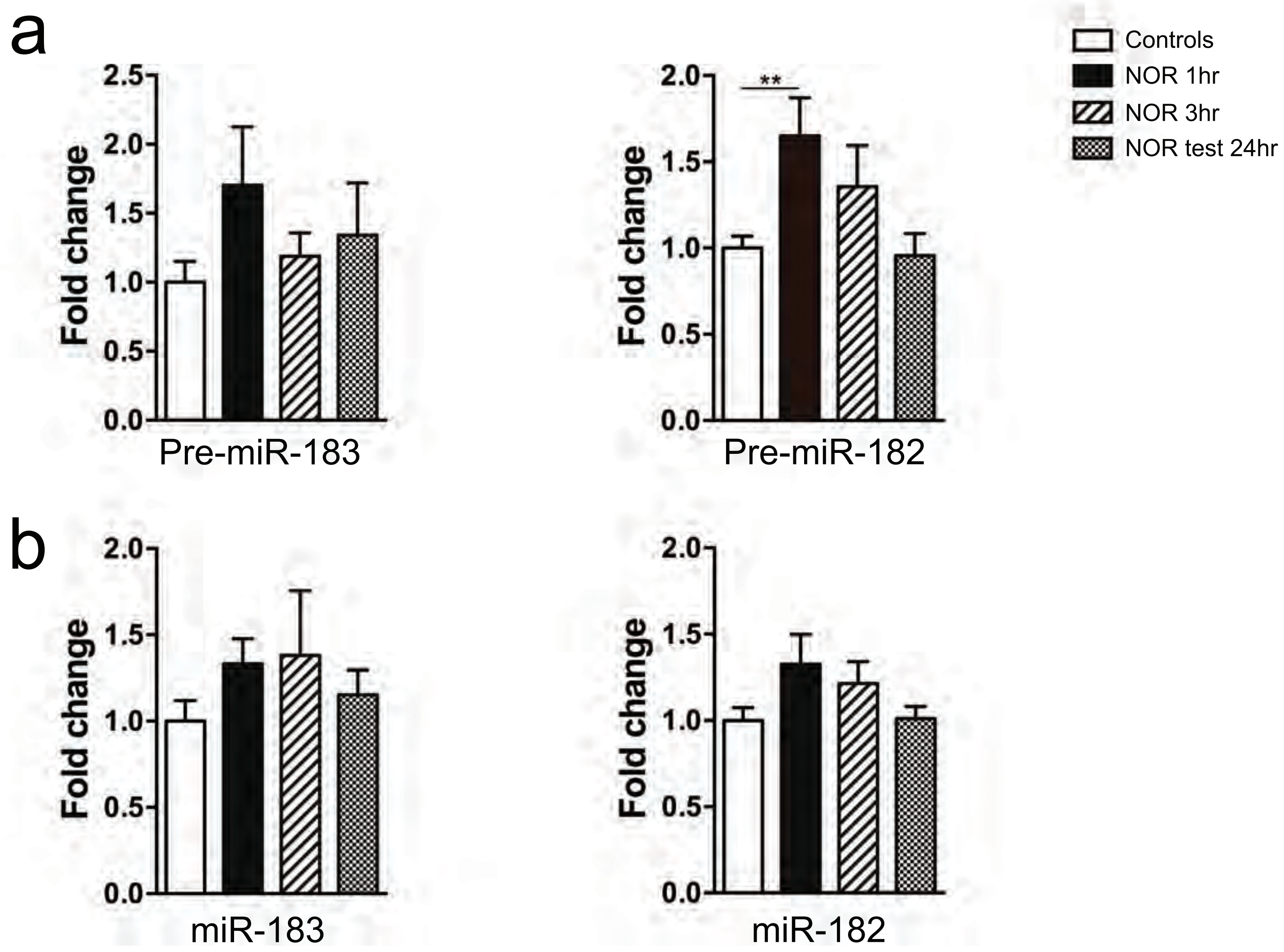


Supplementary Figure 8: MiRNA expression in hippocampus of mice trained with the strong NOR protocol and sacrificed at 1h, 3hr or 24hr after testing. (a) Left panel: pre-miR-183 one-way ANOVA $F_{3,23} = 1.16$, $p=0.35$; right panel: pre-miR-182 one-way ANOVA $F_{3, 23} = 4.00$, $p=0.02$, posthoc (control, NOR 1hr) $^{**}p<0.01$). (b) Left panel: miR-183 one-way ANOVA $F_{3, 23} = 0.78$, $p=0.51$; right panel: miR-182 one-way ANOVA $F_{3, 23} = 2.00$, $p=0.14$. Bar graphs represent mean ± s.e.m.

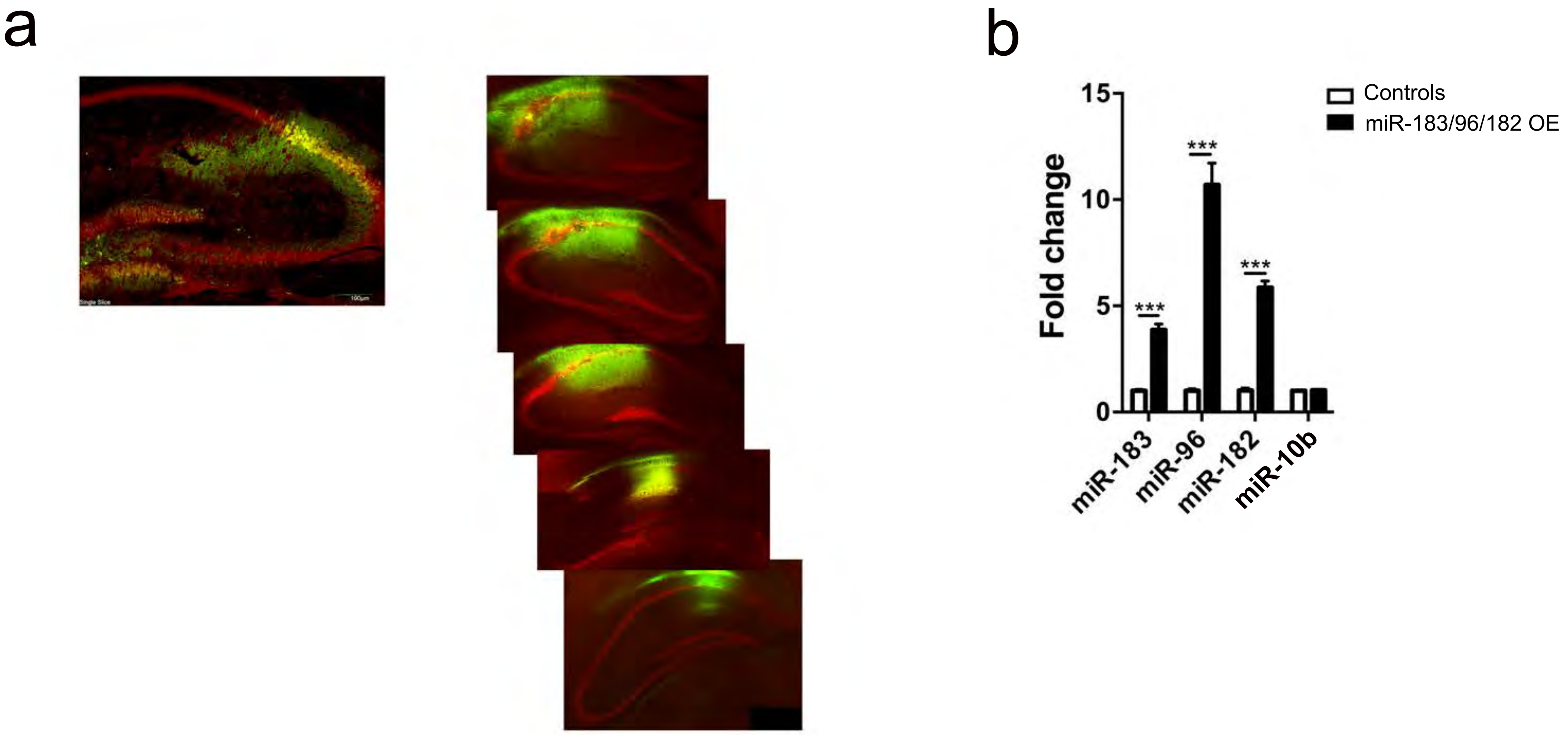


Supplementary Figure 9: AAV-mediated expression of miR-183/96/182 in the mouse hippocampus. (a) GFP staining of a brain slice 21 days after injection of a scAAV-cluster 183-GFP virus into the hippocampus. Neurons are labeled in red and GFP-expressing cells are green (anti-GFP). The series of images shown on the right panel display the extent of spread in the antero-posterior direction. (b) qPCR measurement of mature miR-183/96/182 levels in mouse hippocampus after injection with scAAV-control-GFP or scAAV-cluster 183-GFP (miR-183: $t_4$=10.53, ***$p<0.001$; miR-96, $t_4$=9.43, ***$p<0.001$; miR-182, $t_4$=14.58, ***$p<0.001$; miR-10b, $t_4$=0.55, $p$=0.61; controls, n=3; miR-183/96/182, n=3). Bar graphs represent mean ± s.e.m.

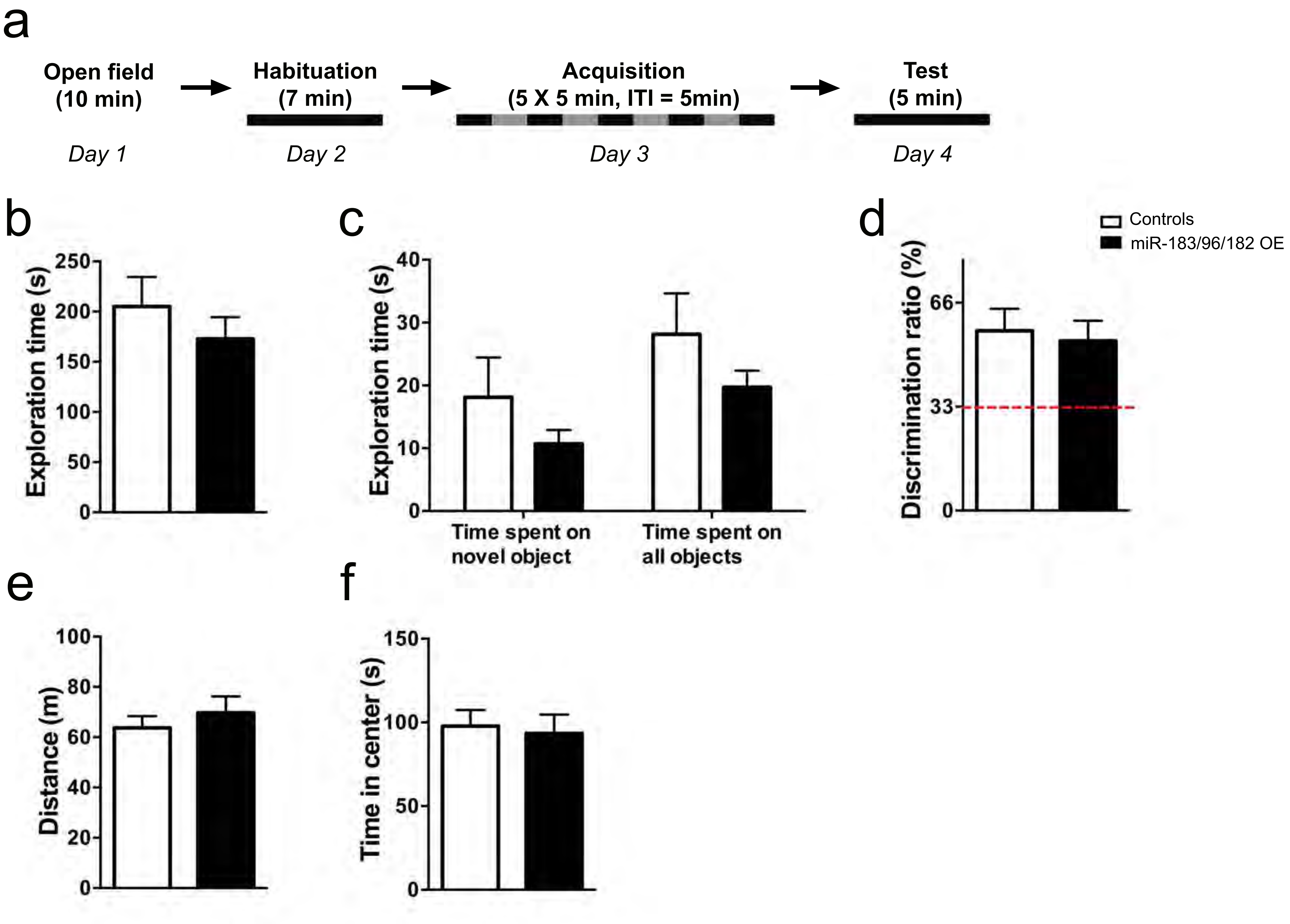
a
Open field
(10 min)
Habituation
(7 min)
Acquisition
(5 X 5 min, ITI = 5min)
Test
(5 min)
Day 1
Day 2
Day 3
Day 4
b
Exploration time (s)
250
200
150
100
50
0
c
Exploration time (s)
40
30
20
10
0
Time spent on
novel object
Time spent on
all objects
d
Controls
miR-183/96/182 OE
Discrimination ratio (%)
66
33
0
e
Distance (m)
100
80
60
40
20
0
f
Time in center (s)
150
100
50
0

Supplementary Figure 10: MiR-183/96/182 overexpression (miR-183/96/182 OE) in the hippocampus does not influence long-term object memory with a strong training protocol. (a) Experimental setup. (b, c) Time spent exploring objects during acquisition (b, $t_{10}=0.88$, $p=0.40$), and testing on the NOR task (c, time on novel object: $t_{10}=1.1$, $p=0.29$; time on all objects: $t_{10}=1.2$, $p=0.26$) (d) Discrimination ratio of novel object over familiar objects (one-sample t-test, control: $t_5=3.43$, $p<0.05$; miR-183/96/182 - $t_5=3.27$, $p<0.05$; unpaired t test between controls and miR-183/96/182: $t_{10}=0.34$, $p=0.74$); the broken line shows chance level of discrimination set at 33%. (e, f) Total distance covered ($t_{10}=0.75$, $p=0.47$) (e), and total time spent in the center of the arena ($t_{10}=0.30$, $p=0.77$) (f), during open field-testing showing similar performance in control and miR-183/96/182 overexpressing mice. Controls, n=6; miR-183/96/182 OE, n=6. Bar graphs represent mean ± s.e.m.

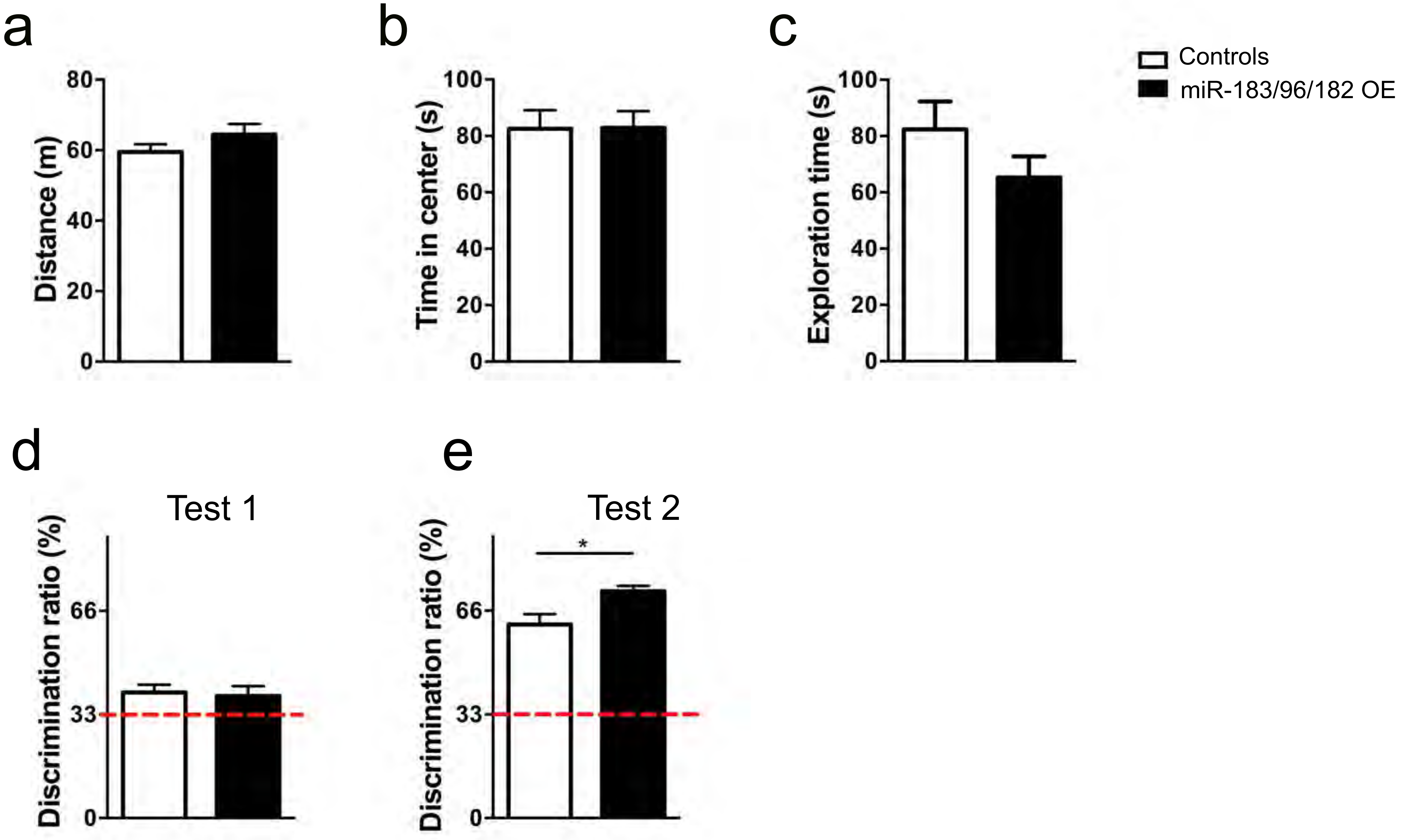


Supplementary Figure 11: Performance of miR-183/96/182 expressing (miR-183/96/182 OE) mice and controls in a weak training protocol. (a) Total path length during open field test ($t_{32}$=1.30, p=0.20); (b) Time spent in the center of the arena during open field test ($t_{32}$=0.04, p=0.97); (c)Time spent exploring objects during acquisition ($t_{32}$=1.32, p=0.197). (d) Discrimination ratio during test 1, 24hr after NOR training (One sample t-test, controls: $t_{18}$=2.86, $p<0.05$; miR-183/96/182, $t_{14}$=1.84, $p<0.1$); unpaired t-test between group comparison: ($t_{32}$=0.3, p=0.77). (e) Discrimination ratio during test 2, 48hr after NOR training (One sample t-test, control: $t_{18}$=8.82, $p<0.001$; miR-183/96/182: $t_{14}$=22.37, $p<0.001$); unpaired t-test between group comparison ($t_{32}$=2.65, *$p<0.05$). Controls, n=19; miR-183/96/182 OE, n=15. Bar graphs represent mean ± s.e.m.

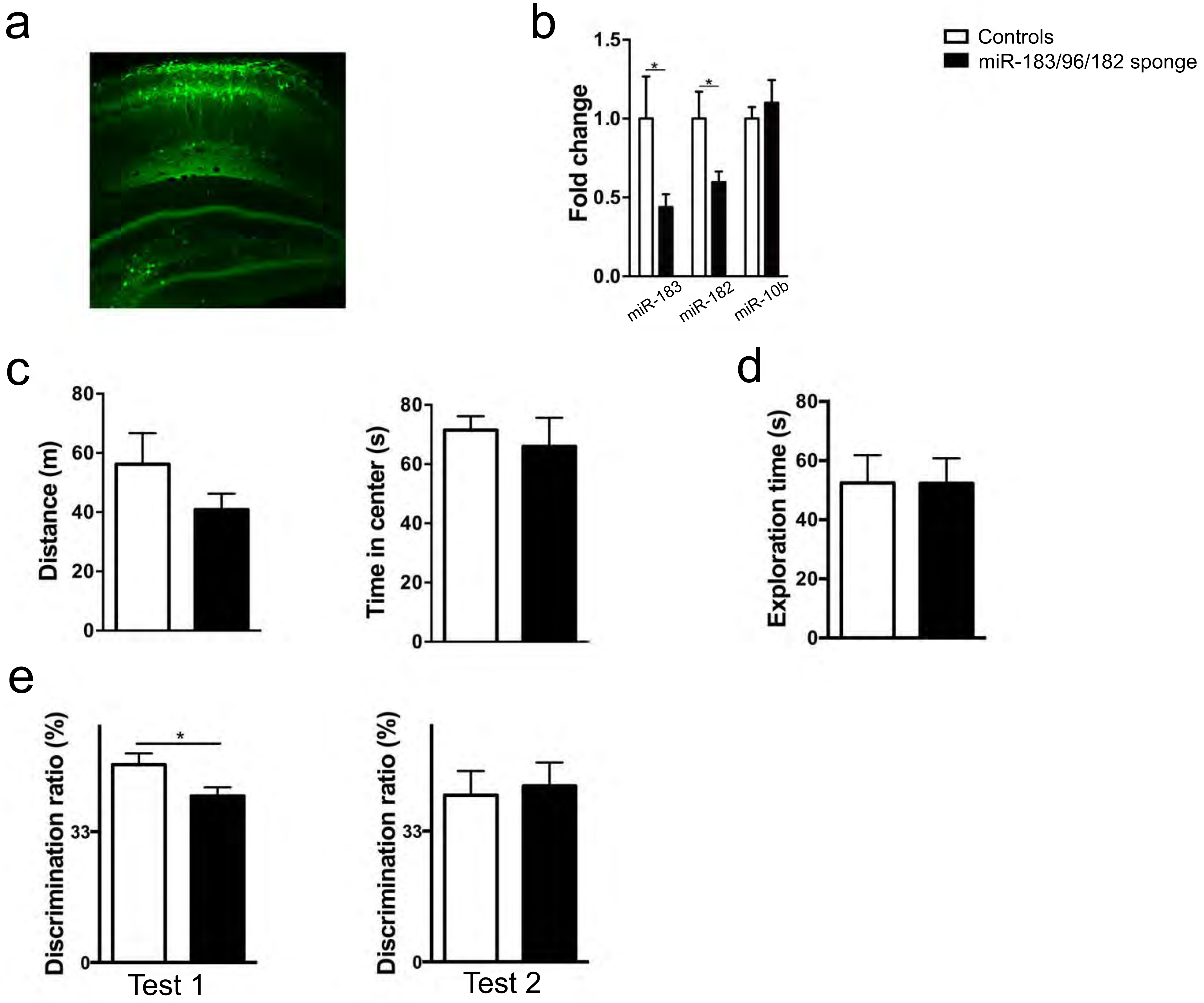
a
b
Controls
miR-183/96/182 sponge
Fold change
1.5
1.0
0.5
0.0
*
*
miR-183
miR-182
miR-10b
c
Distance (m)
80
60
40
20
0
Time in center (s)
80
60
40
20
0
d
Exploration time (s)
80
60
40
20
0
e
Discrimination ratio (%)
33
0
*
Test 1
Discrimination ratio (%)
33
0
Test 2

Supplementary Figure 12. Performance of mice expressing miR-183/96/182 sponge in the hippocampus under the weak NOR paradigm. (a) GFP expression in CA1 area of the hippocampus 10 days after scAAV-hSyn-miR-183 cluster sponge injection. (b) Quantification of miRNA expression in the hippocampus of mice expressing the sponge construct 30 days after virus injection (miR-183: $t_{14}=2.23$, *$p<0.05$; miR-182: $t_{14}=2.40$, *$p<0.05$; miR-10b: $t_{14}=0.57$, $p=0.58$); Control, n=7, miR-183/96/182, n=9. (c) Total distance covered (left panel: $t_{18}=1.31$, $p=0.21$) and time spent in center arena (right panel: $t_{18}=0.52$, $p=0.609$) during open-field test. (d) Total object exploration during 10min acquisition session ($t_{18}=0.01$, $p=0.99$). (e) Discrimination ratio 24hr (test 1: $t_{17}=0.25$, *$p<0.5$) and 48hr (test 2, $t_{16}=0.28$, $p=0.78$) after NOR training. Control, n=9-10; miR-183/96/182 sponge, n=9-10). Bar graphs represent mean ± s.e.m.

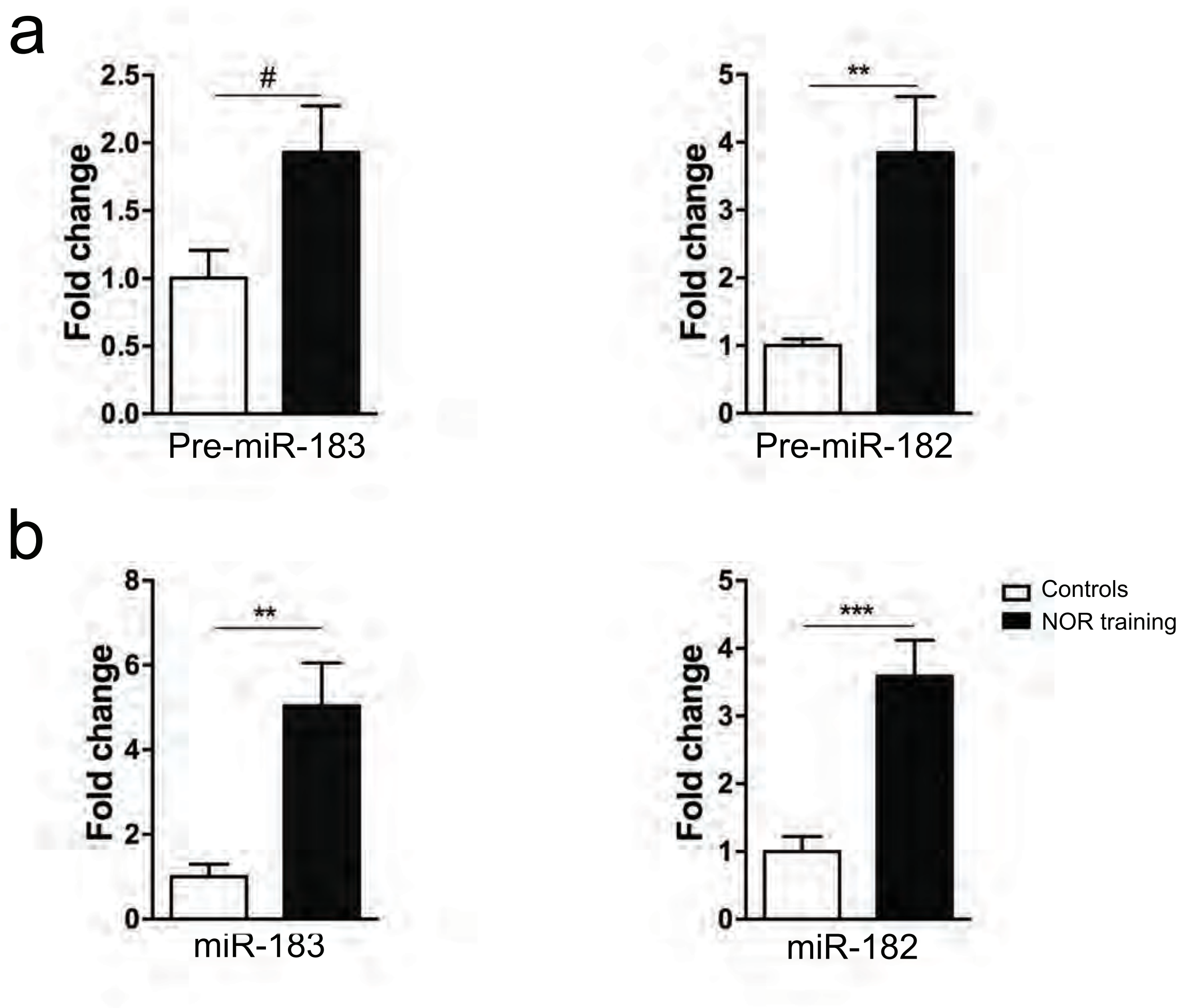


Supplementary Figure 13: Hippocampal miRNA expression 30 minutes after training with the weak NOR protocol. (a) Left panel: pre-miR-183, $t6=2.32$, #$p=0.06$; right panel: pre-miR-182, $t7=3.01$, *$p=0.02$. (b) Left panel: miR-183, $t9=4.15$, **$p<0.01$; right panel: miR-182, $t9=4.8$, ***$p\leq0.001$. Control, n= 4-6; NOR, n=5. Bar graphs represent mean ± s.e.m.

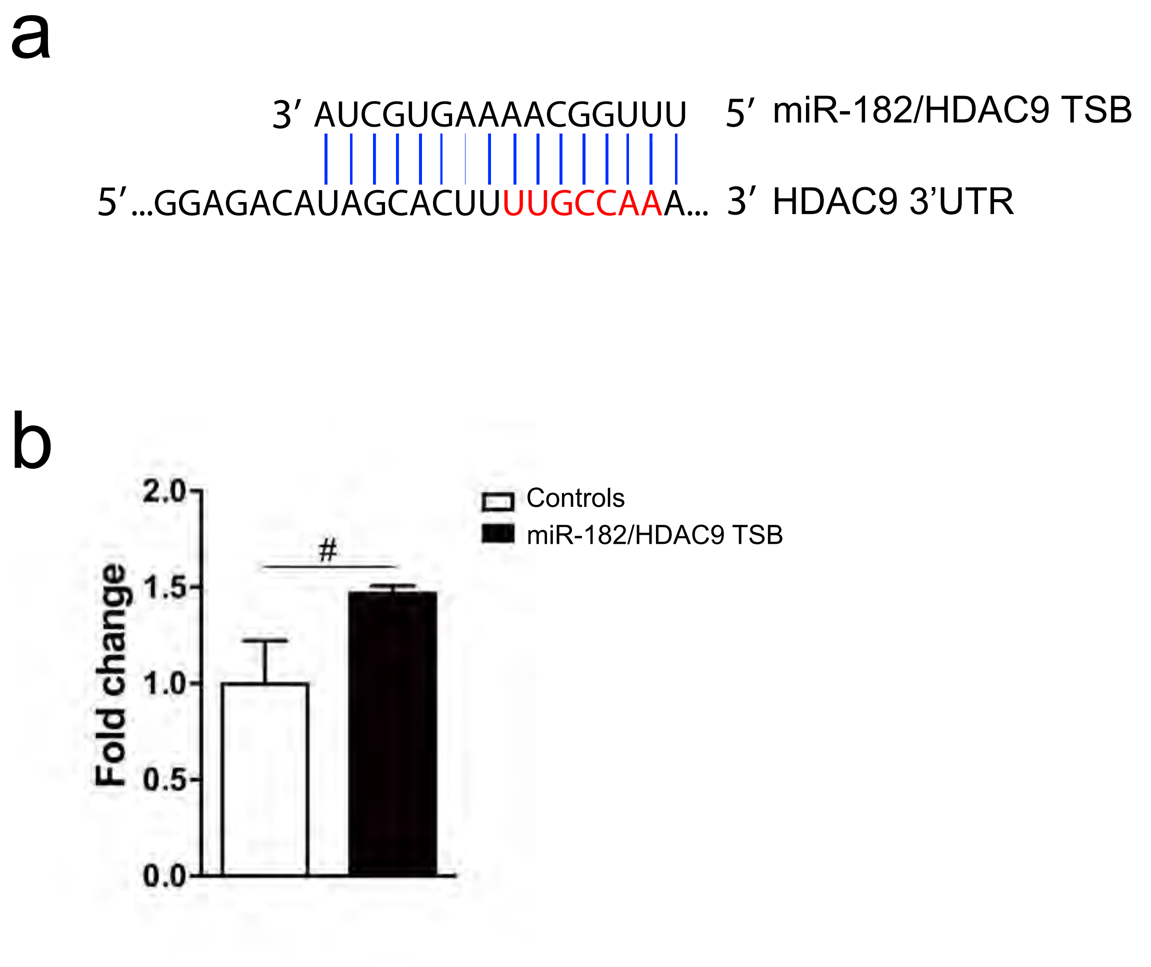


Supplementary Figure 14: miR-182/HDAC9 target site blocker (TSB) and its effect on HDAC9 expression. (a) Alignment of miR-182 TSB at HDAC9 3’UTR; sequences labeled in red are miR-182 seed sequence binding sites. (b) HDAC9 mRNA level in N2A cells treated with miR-182 TSB ($t_6$=2.05, #p=0.087). Bar graphs represent mean ± s.e.m.

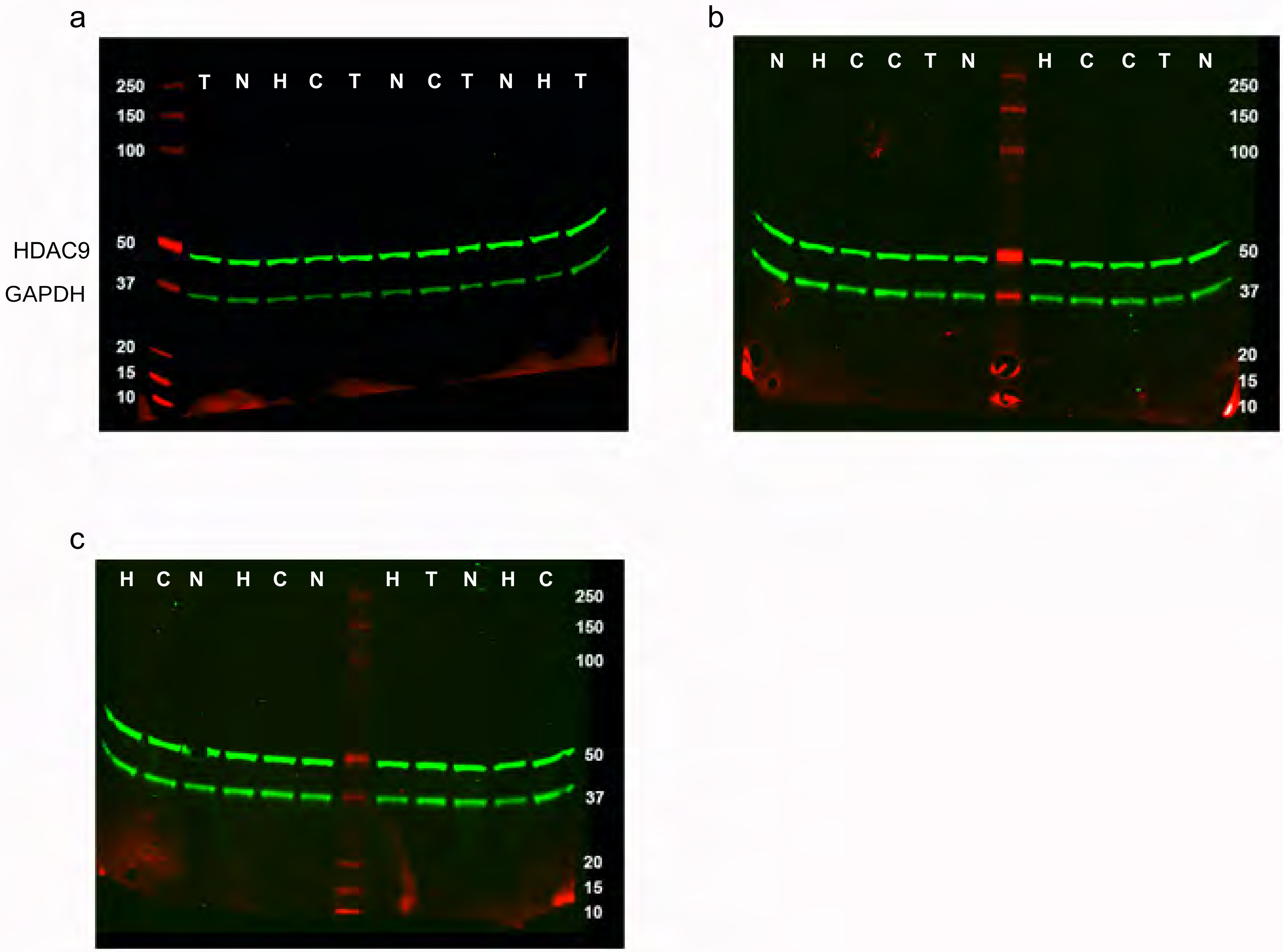


Supplementary Figure 15: Western blots for HDAC9 and GAPDH on hippocampal extracts (a-c). The numbers on the sides refer to molecular weight of the marker. C: cage control, H: habituation only, N: NOR training, T: training and test.

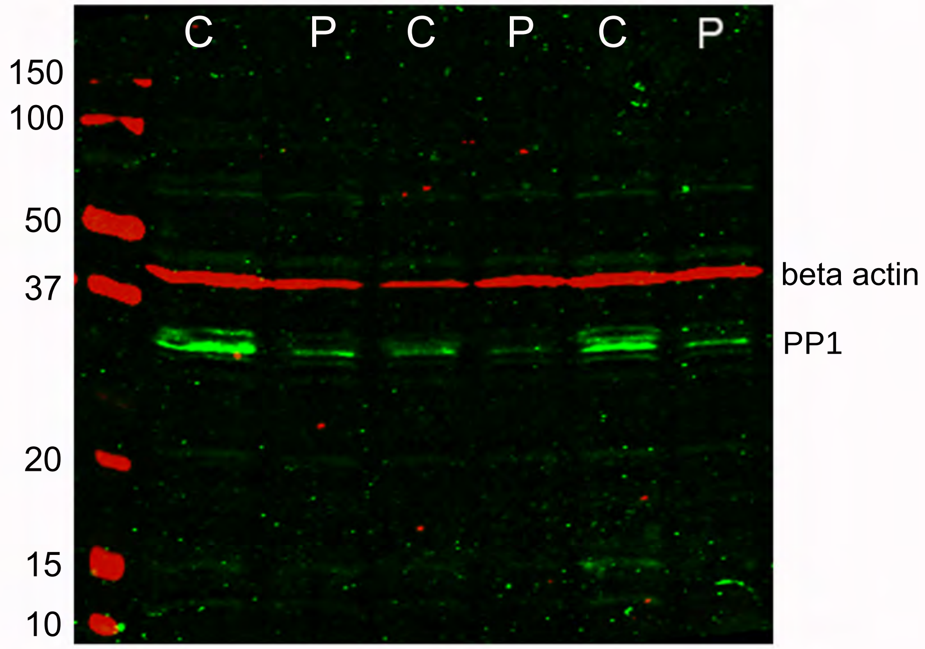


Supplementary Figure 16: Western blots for PP1 and beta actin on whole cell lysate from N2A cells transfected with PP1g siRNA (P) or scrambled control siRNA (C). The numbers on the side indicate molecular weight of the marker.

(a)

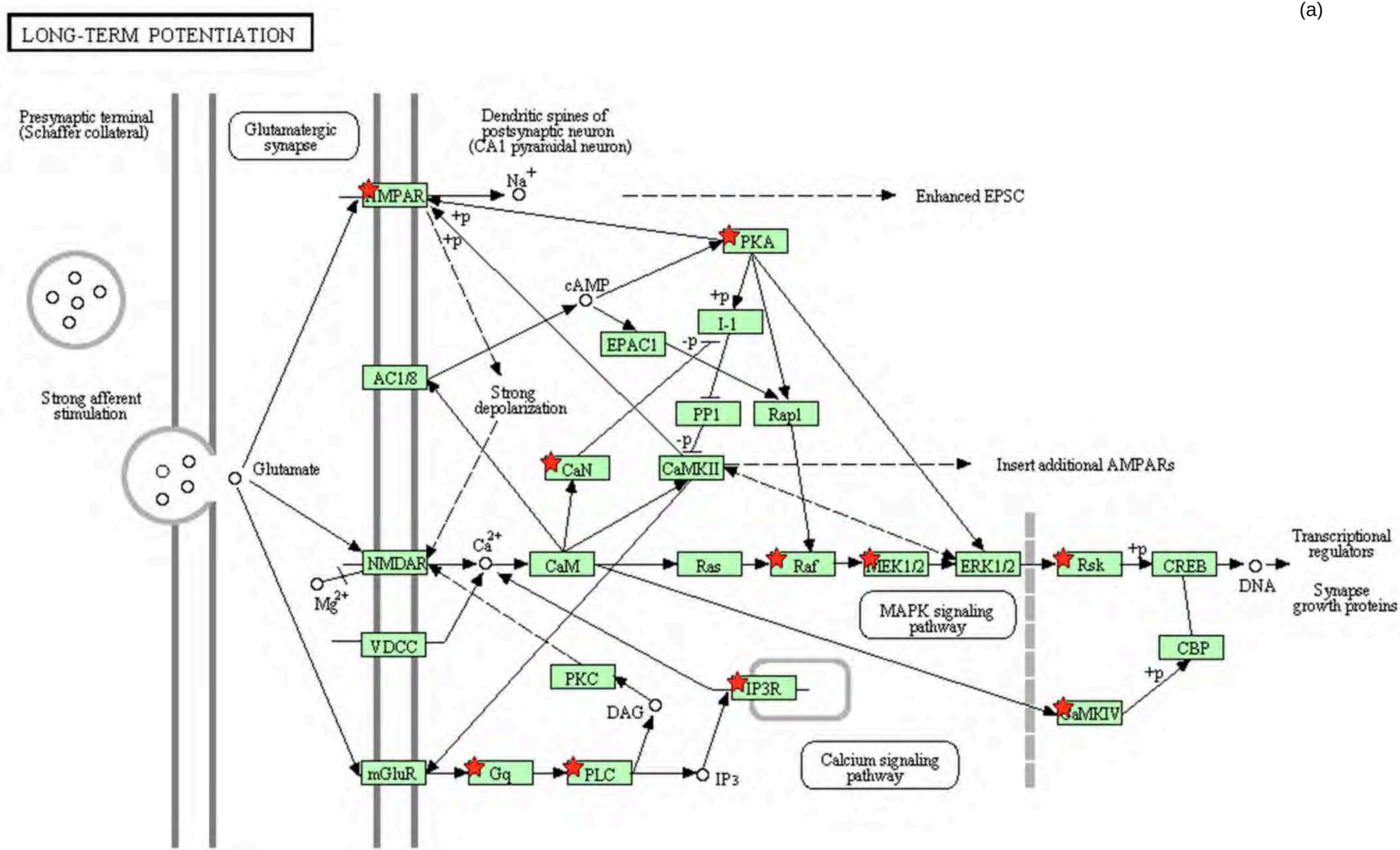

LONG-TERM POTENTIATION
Presynaptic terminal (Schaffer collateral)
Glutamatergic synapse
Dendritic spines of postsynaptic neuron (CA1 pyramidal neuron)
AMPAR
Na+
Enhanced EPSC
+p
PKA
cAMP
I-1
EPAC1
-p
AC1/8
Strong depolarization
PP1
Rap1
Strong afferent stimulation
CaN
CaMKII
Insert additional AMPARs
Glutamate
Ca2+
NMDAR
CaM
Ras
Raf
MEK1/2
ERK1/2
Rsk
CREB
DNA
Transcriptional regulators
Synapse growth proteins
Mg2+
MAPK signaling pathway
VDCC
CBP
PKC
IP3R
DAG
CaMKIV
mGluR
Gq
PLC
IP3
Calcium signaling pathway

(b)

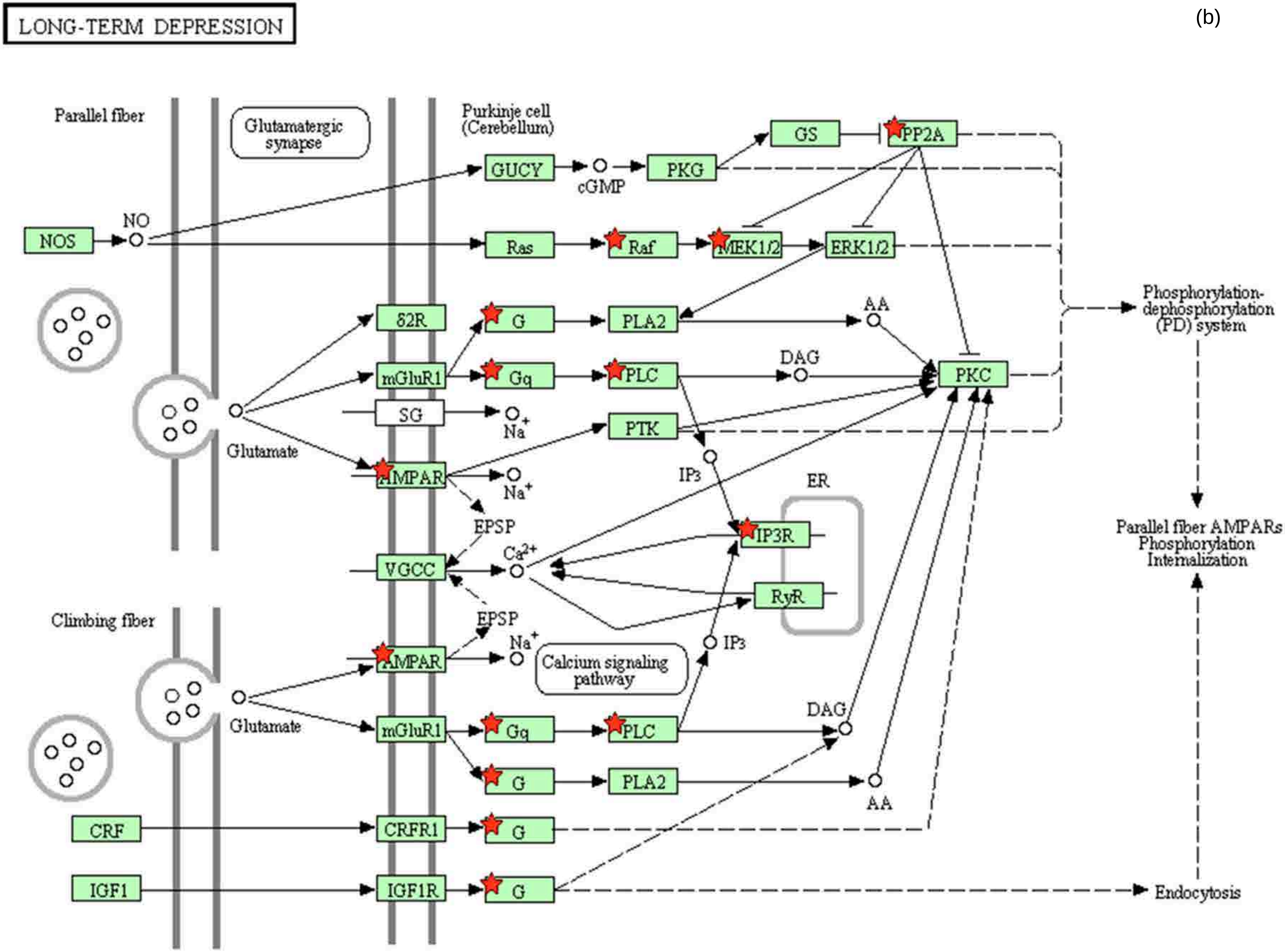

LONG-TERM DEPRESSION
Parallel fiber
Glutamatergic synapse
Purkinje cell (Cerebellum)
GUCY
cGMP
PKG
GS
PP2A
NOS
NO
Ras
Raf
MEK1/2
ERK1/2
Phosphorylation-dephosphorylation (PD) system
δ2R
G
PLA2
AA
mGluR1
Gq
PLC
DAG
PKC
SG
Na+
PTK
Glutamate
AMPAR
Na+
IP3
ER
EPSP
IP3R
VGCC
Ca2+
Parallel fiber AMPARs Phosphorylation Internalization
RyR
Climbing fiber
EPSP
Na+
IP3
AMPAR
Calcium signaling pathway
Glutamate
mGluR1
Gq
PLC
DAG
G
PLA2
AA
CRF
CRFR1
G
IGF1
IGF1R
G
Endocytosis

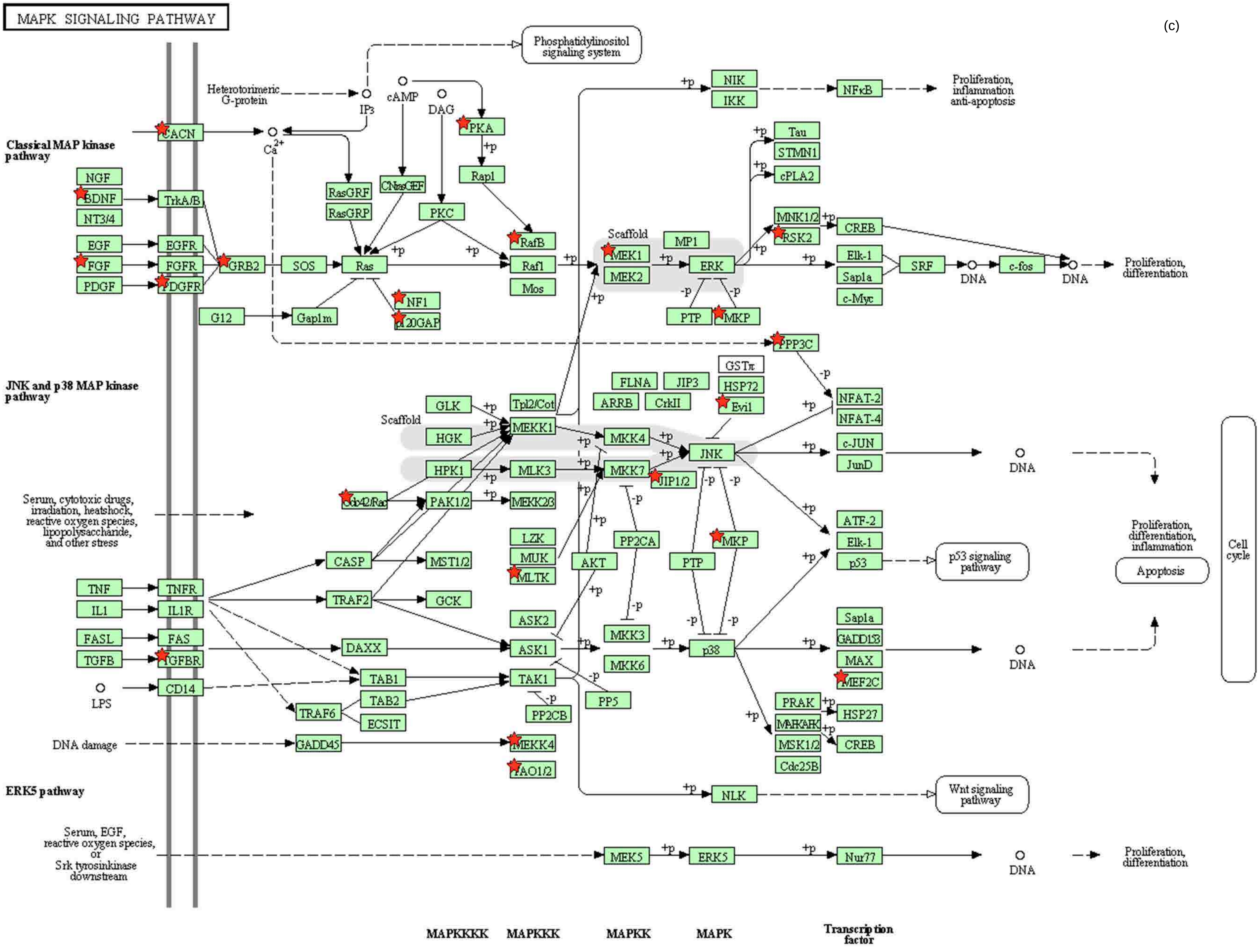

(c)
MAPK SIGNALING PATHWAY
Classical MAP kinase pathway
JNK and p38 MAP kinase pathway
ERK5 pathway
Heterotorimeric G-protein
Phosphatidylinositol signaling system
IP3
cAMP
DAG
Ca2+
CACN
PKA
Rap1
RasGRF
RasGRP
CNrasGEF
PKC
NGF
BDNF
NT3/4
TrkA/B
EGF
EGFR
FGF
FGFR
PDGF
PDGFR
GRB2
SOS
Ras
RafB
Raf1
Mos
G12
Gap1m
NF1
p120GAP
Scaffold
MEK1
MEK2
MP1
ERK
PTP
MKP
NIK
IKK
NFκB
Proliferation, inflammation anti-apoptosis
Tau
STMN1
cPLA2
MNK1/2
RSK2
CREB
Elk-1
Sap1a
c-Myc
SRF
c-fos
DNA
Proliferation, differentiation
PPP3C
NFAT-2
NFAT-4
GSTπ
HSP72
Evi1
FLNA
JIP3
ARRB
CrkII
GLK
HGK
HPK1
Tpl2/Cot
MEKK1
MLK3
MKK4
MKK7
JIP1/2
JNK
c-JUN
JunD
Serum, cytotoxic drugs, irradiation, heatshock, reactive oxygen species, lipopolysaccharide, and other stress
Cdc42/Rac
PAK1/2
MEKK2/3
LZK
MUK
MLTK
AKT
PP2CA
ATF-2
p53
p53 signaling pathway
Proliferation, differentiation, inflammation
Apoptosis
Cell cycle
TNF
TNFR
IL1
IL1R
FAS
FASL
TGFB
TGFBR
LPS
CD14
CASP
MST1/2
TRAF2
GCK
DAXX
ASK2
ASK1
MKK3
MKK6
p38
Sap1a
GADD153
MAX
MEF2C
TAB1
TAB2
TAK1
TRAF6
ECSIT
PP2CB
PP5
PRAK
MAPKAPK
MSK1/2
Cdc25B
HSP27
DNA damage
GADD45
MEKK4
TAO1/2
NLK
Wnt signaling pathway
Serum, EGF, reactive oxygen species, or Srk tyrosinkinase downstream
MEK5
ERK5
Nur77
MAPKKKK
MAPKKK
MAPKK
MAPK
Transcription factor

(d)

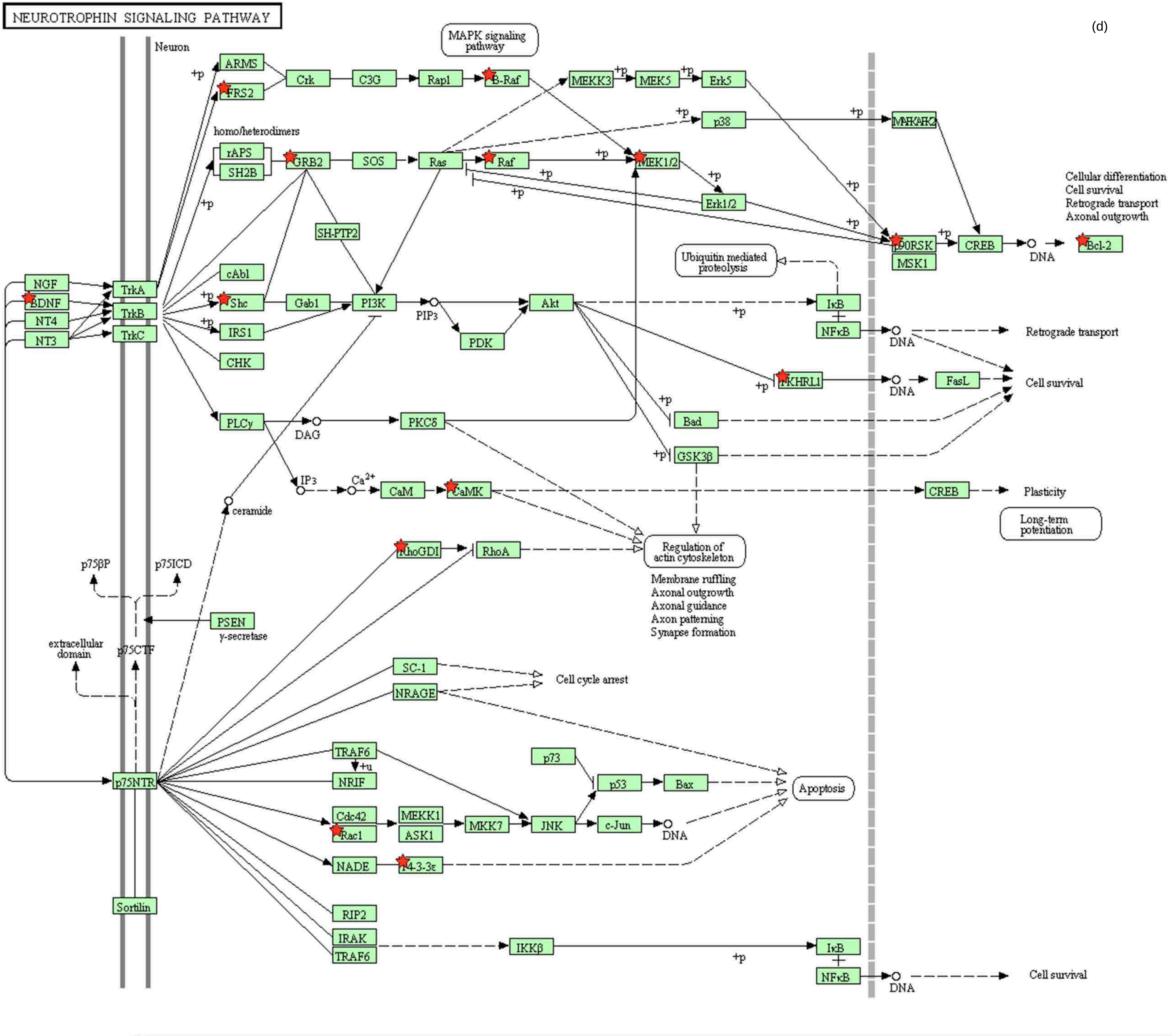

NEUROTROPHIN SIGNALING PATHWAY
Neuron
MAPK signaling pathway
ARMS
FRS2
Crk
C3G
Rap1
B-Raf
MEKK3
MEK5
Erk5
homo/heterodimers
rAPS
SH2B
GRB2
SOS
Ras
Raf
MEK1/2
p38
MAPKAPK2
Erk1/2
SH-PTP2
p90RSK
MSK1
CREB
DNA
Bcl-2
Cellular differentiation
Cell survival
Retrograde transport
Axonal outgrowth
Ubiquitin mediated proteolysis
NGF
BDNF
NT4
NT3
TrkA
TrkB
TrkC
cAbl
Shc
Gab1
PI3K
PIP3
Akt
IκB
NFκB
IRS1
PDK
Retrograde transport
Cell survival
CHK
PKHRL1
FasL
PLCγ
DAG
PKCδ
Bad
GSK3β
IP3
Ca2+
CaM
CaMK
CREB
Plasticity
Long-term potentiation
ceramide
RhoGDI
RhoA
Regulation of actin cytoskeleton
Membrane ruffling
Axonal outgrowth
Axonal guidance
Axon patterning
Synapse formation
p75βP
p75ICD
PSEN
γ-secretase
extracellular domain
p75CTF
SC-1
NRAGE
Cell cycle arrest
TRAF6
NRIF
p73
p53
Bax
Apoptosis
p75NTR
Cdc42
Rac1
MEKK1
ASK1
MKK7
JNK
c-Jun
NADE
14-3-3ε
Sortilin
RIP2
IRAK
TRAF6
IKKβ
IκB
NFκB
Cell survival

(e)

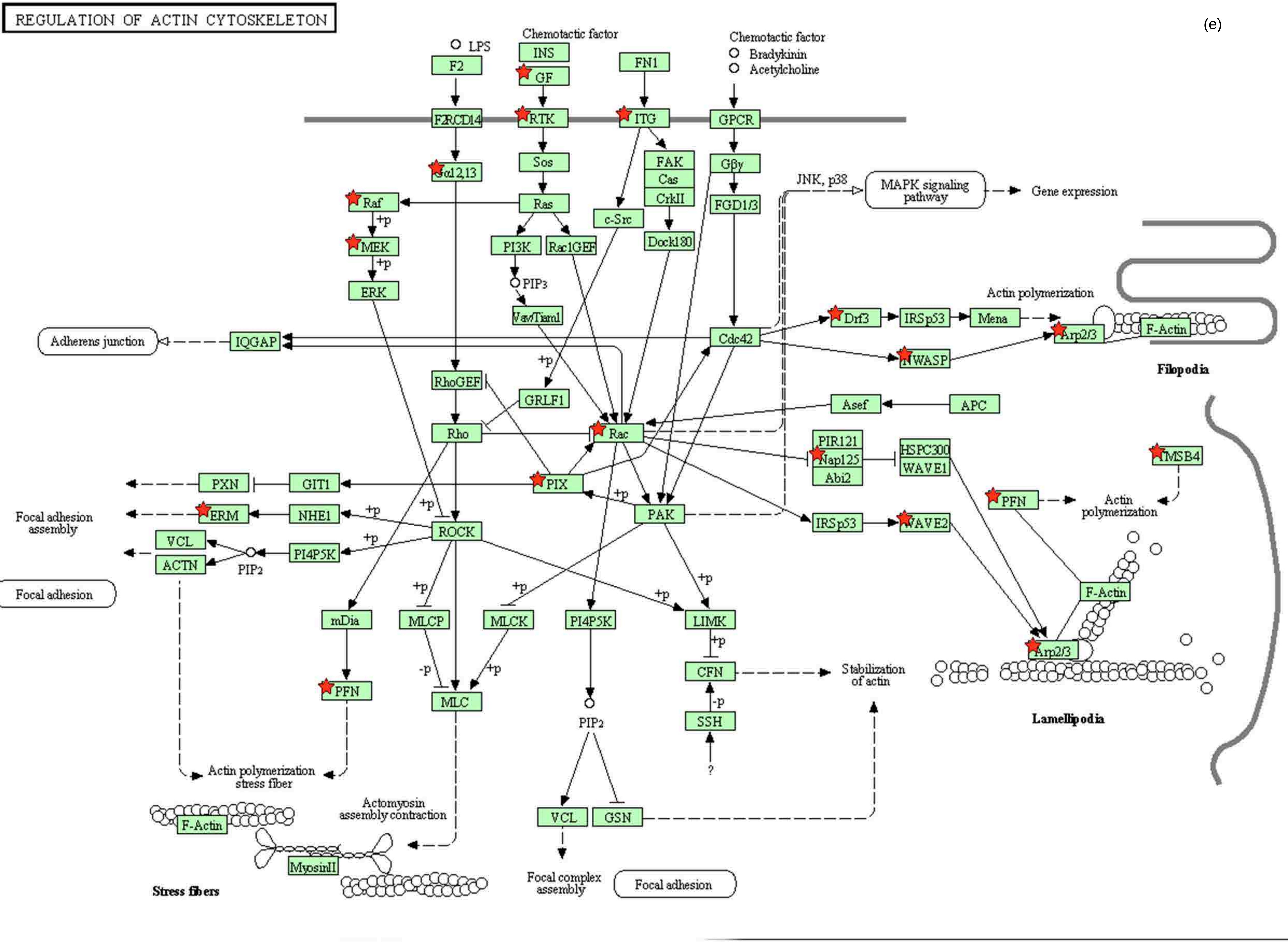
REGULATION OF ACTIN CYTOSKELETON
LPS
F2
Chemotactic factor
INS
GF
FN1
Chemotactic factor
Bradykinin
Acetylcholine
F2RCD14
RTK
ITG
GPCR
Gα12,13
Sos
FAK
Cas
CrkII
Gβγ
JNK, p38
MAPK signaling pathway
Gene expression
Raf
Ras
c-Src
FGD1/3
+p
MEK
PI3K
Rac1GEF
Dock180
+p
ERK
PIP3
Actin polymerization
VavTiam1
Drf3
IRSp53
Mena
Adherens junction
IQGAP
Cdc42
Arp2/3
F-Actin
NWASP
+p
Filopodia
RhoGEF
GRLF1
Asef
APC
Rho
Rac
PIR121
Nap125
Abi2
HSPC300
WAVE1
TMSB4
PXN
GIT1
PIX
+p
PFN
Actin polymerization
Focal adhesion assembly
ERM
NHE1
+p
+p
PAK
IRSp53
WAVE2
ROCK
+p
VCL
ACTN
PIP2
PI4P5K
Focal adhesion
F-Actin
+p
+p
+p
+p
+p
mDia
MLCP
MLCK
PI4P5K
LIMK
+p
Arp2/3
-p
+p
CFN
Stabilization of actin
PFN
MLC
PIP2
-p
SSH
Lamellipodia
?
Actin polymerization stress fiber
Actomyosin assembly contraction
F-Actin
VCL
GSN
MyosinII
Stress fibers
Focal complex assembly
Focal adhesion

Supplementary Figure 17: Top pathways with predicted target genes of miR-183/182/96 cluster. Many predicted target genes (highlighted with red stars) are involved in regulation of long-term potentiation (a), long-term depression (b), MAPK signaling (c), neurotrophin signaling (d), and actin cytoskeleton (e). Target prediction and pathway analysis performed using mirPath and DAVID respectively and schematics was generated using KEGG pathway database.

**Supplementary Table 1: List of primers, siRNAs, and plasmids**

| Primer/siRNA/plasmid | Source/sequence |
|---|---|
| miR-183, forward primer | Qiagen, cat. No.: MS00001722 |
| miR-182, forward primer | Qiagen, cat. No.: MS00011291 |
| miR-96, forward primer | Qiagen, cat. No.: MS00001456 |
| miR-135a, forward primer | Qiagen, cat. No.: MS00011130 |
| miR-135b, forward primer | Qiagen, cat. No.: MS00001575 |
| miR-204-3p, forward primer | Qiagen, cat. No.: MS00024514 |
| miR-146, forward primer | Qiagen, cat. No.: MS00001638 |
| miR-200c, forward primer | Qiagen, cat. No.: MS00001827 |
| Let-7c, forward primer | Qiagen, cat. No.: MS00005852 |
| miR-190, forward primer | Qiagen, cat. No.: MS00032438 |
| Pre-miR-10b, forward primer | Qiagen, cat. No.: MP00003983 |
| miR-10b, forward primer | Qiagen, cat. No.: MS00032249 |
| RNU6, forward primer | Qiagen, cat. No.: MS00033740 |
| 10X miScript Universal reverse primer | Qiagen, cat. No.: 218193 |
| Pre-miR-96, forward & reverse primers | Qiagen, cat. No.: MP00006881 |
| Pre-miR-183, forward & reverse primers | Qiagen, cat. No.: MP00004438 |
| Pre-miR-182, forward & reverse primers | Qiagen, cat. No.: MP00004431 |
| Pri-miR-183/96/182 forward | CCC TCC TAA AAC CAC CCT AA |
| Pri-miR-183/96/182 reverse | AGT TGG CAA GTC TAG AAC CAC |
| HDAC9, forward primer | ACG AGA AAG GGC AGT GGC AAG C |
| HDAC9, reverse primer | GAT GTG TGG TGG GCA GCC GT |
| NUFIP2, forward primer | AAC GCC GAA GAA GAA AAC AGG CTA |
| NUFIP2, reverse primer | GCT GAC ATC GGG ACC TGG GA |
| GAPDH, forward primer | CCA CTG GTG CTG CCA AGG CT |
| GAPDH, reverse primer | GGC AGG TTT CTC CAG GCG GC |
| Cacnb4, forward primer | TAC CTG CAT GGA GTT GAA GAC T |
| Cacnb4, reverse primer | TTC GCT CTC TCA AGC TGG ATA |
| Gabra1, forward primer | TGT GCG AGG GAG AGC AAG TC |
| Gabra1, reverse primer | AGC TAG GAA GCA GGG AGA TGT A |
| Prkcz, forward primer | GAC TGG GTG CAG ACA GAG AAA C |
| Prkcz, reverse primer | ACT CGA TGA CCA GGA ACA ACC G |
| 18s rRNA, forward primer | CGGCTACCACATCCAAGGAA |
| 18s rRNA, reverse primer | GCTGGAATTACCGCGGCT |
| Tubd1, forward primer | TCTCTTGCTAACTTGGTGGTCCTC |
| Tubd1, reverse primer | GCTGGGTCTTTAAATCCCTCTACG |
| Nrg1, forward primer | TGT GGT GGC CTA CTG CAA AA |
| Nrg1, reverse primer | TGG TGG GTT TGG ATG GTG AG |
| Ppp2ca, forward primer | ATG GAC GAG AAG TTG TTC ACC |
| Ppp2ca, reverse primer | CAG TGA CTG GAC ATC GAA CCT |
| Grm5, forward primer | CGT CTG GGG AAA CCC TAA GCT CCA |
| Grm5, reverse primer | TCA CCT CGA TGG CCG GCA GA |
| Gria1, forward primer | GTC CGC CCT GAG AAA TCC AG |
| Drosha, forward primer | GAG CCT AGA GGA AGC CAA ACA |
| Drosha, reverse primer | GCC GGA CGT GAG TGA AGA T |
| Dicer, forward primer | TTA ACC TTT TGG TGT TTG ATG AGT GT |
| Dicer, reverse primer | GCG AGG ACA TGA TGG ACA ATT |
| Ago2, forward primer | CCA TCT AGC TGT GAA GGC TCT GA |
| Ago2, reverse primer | TTC TTA GGG CCA GGC TTT AAA A |
| Gria1, reverse primer | CTC GCC CTT GTC GTA CCA C |
| Usp13, forward primer | CCC AGG GTA CAC GGG CCT GA |
| Usp13, reverse primer | GCT GAG GCT TGT GCT CCT CCT TC |
| Tubd1, forward primer | GGG AGA ATC ATG GAC CAG AA |
| Tubd1, reverse primer | TTG CTG CTG CTG TCT TTG TT |
| PP1γ siRNA pool | ON-TARGETplus Mouse Ppp1cc (19047) siRNA-SMARTpool, 5nmol. ThermoScientific catalog number L-040212-00-0005 |
| Negative control siRNA | AllStars Negative control siRNA (20 nmol). Qiagen, catalog Number 1027281 |
| PP1γ 3' UTR plasmid | Ppp1cc (GFP-tagged) - Mouse protein phosphatase 1, catalytic subunit, gamma isoform (cDNA clone MGC:13976 IMAGE:3487479), (10ug), 10µg. Origene, catalog number: MG204669 |

**Supplementary Table 2: Results from t-tests, Fisher's LSD (protected or unprotected) and Tukey's posthoc analyses when appropriate.**

| Figure number | Description | t-test | One- or two-way ANOVA | Fisher's LSD posthoc | Tukey's posthoc |
|---|---|---|---|---|---|
| 2c, left | Effect of PP1γ knockdown on pre-miR-183 | Control vs PP1γ k/d t(4)=3.09, p=0.037 | F(2,6)=3.12, p=0.12 | t(6)=2.43, p=0.05 | n/a |
| 2c, middle | Effect of PP1γ knockdown on pre-miR-96 | Control vs PP1γ k/d t(4)=3.75, p=0.02 | F(2,6)=9.19, p=0.01 | t(6)=3.83, p=0.009 | n/a |
| 2c, right | Effect of PP1γ knockdown on pre-miR-182 | Control vs PP1γ k/d t(4)=3.52, p=0.02 | F(2,6)=4.13, p=0.07 | t(6)=2.82, p=0.03 | n/a |
| 2d | Effect of PP1γ knockdown on pri-miR-183/96/182 | | PP1 F(1,8)=5.37, p=0.049<br><br>ActD F(1,8)=35.62, p=0.0003 | Vehicle t(8)= 3.30, p=0.01<br><br>ActD t(8)=0.02, p=0.99 | Vehicle q(8)=4.66, p=0.04<br><br>ActD q(8)=0.02, p>0.99 |
| 2e, left | pre-miR-183 production with PP1 inhibition and ActD | Vehicle t(16)=3.47, p=0.004<br><br>ActD t(11)=2.53, p=0.028 | PP1 F(1,27)=17.61, p=0.0003 | Vehicle t(27)=3.13, p=0.004<br><br>ActD t(27)=2.85, p=0.008 | Vehicle q(27)=4.43, p=0.02<br><br>ActD q(27)=4.02, p=0.04 |
| 2e, middle | pre-miR-96 production with PP1 inhibition and ActD | Vehicle t(16)=3.97, p=0.001<br><br>ActD t(27)=1.89, p=0.08 | PP1 F(1,27)=16.13, p=0.0004 | Vehicle t(27)=4.05, p=0.0004<br><br>ActD t(27)=1.80, p=0.078 | Vehicle q(27)=5.73, p=0.002<br><br>ActD q(27)=2.59, p=0.28 |
| 2e, right | pre-miR-182 production with PP1 inhibition and ActD | Vehicle t(16)=4.80, p=0.0002<br><br>ActD t(12)=2.43, p=0.03 | PP1 F(1,28)=21.3, p<0.0001 | Vehicle t(28)=3.45, p=0.002<br><br>ActD t(28)=3.11, p=0.004 | Vehicle q(28)=4.88, p=0.009<br><br>ActD q(28)=4.40, p=0.02 |
| 6c | HDAC9 protein level after NOR training | Cage control vs NOR testing t(14)=2.05, p=0.06<br><br>Habituation vs NOR testing t(13)=2.24, p=0.04 | F(3,28)=2.06, p=0.13 | Cage control vs NOR testing t(28)=2.20, p=0.036<br><br>Habituation vs NOR testing t(28)=1.99, p=0.05 | n/a |
| Suppl. 7 | Effect of ActD treatment on C-fos expression | | ActD F(1,8)=29.77, p=0.0006 | Control t(8)=3.86, p=0.0048<br><br>PP1γ k/d t(8)=3.86, p=0.0048 | Control t(8)=5.46, p=0.02<br><br>PP1γ k/d t(8)=5.45, p=0.02 |
| Suppl. 8a, left | Pre-miR-183 expression after training with strong NOR protocol | | F(3,23)=1.16, p=0.35 | n/a | n/a |
| Suppl. 8a right | Pre-miR-182 expression after training with strong NOR protocol | | F(3,23)=4.00, p=0.02 | Control vs NOR 1hr t(23)=2.97, p=0.007 | Control vs NOR 1hr q(23)=4.20, p=0.03 |
| Suppl. 8b, left | miR-183 expression after training with strong NOR protocol | | F(3,23)=0.78, p=0.51 | n/a | n/a |
| Suppl. 8b, right | miR-182 expression after training with strong NOR protocol | | F(3,23)=2.00, p=0.14 | n/a | n/a |
